# Winter Temperature Affects the Prevalence of Ticks in an Arctic Seabird

**DOI:** 10.1371/journal.pone.0065374

**Published:** 2013-06-04

**Authors:** Sébastien Descamps

**Affiliations:** Norwegian Polar Institute, Fram Centre, Tromsø, Norway; Phillip Island Nature Parks, Australia

## Abstract

The Arctic is rapidly warming and host-parasite relationships may be modified by such environmental changes. Here, I showed that the average winter temperature in Svalbard, Arctic Norway, explained almost 90% of the average prevalence of ticks in an Arctic seabird, the Brünnich’s guillemot *Uria lomvia*. An increase of 1°C in the average winter temperature at the nesting colony site was associated with a 5% increase in the number of birds infected by these ectoparasites in the subsequent breeding season. Guillemots were generally infested by only a few ticks (≤5) and I found no direct effect of tick presence on their body condition and breeding success. However, the strong effect of average winter temperature described here clearly indicates that tick-seabird relationships in the Arctic may be strongly affected by ongoing climate warming.

## Introduction

Changes in climate, including regional increases in temperatures, are altering the structure and function of ecosystems globally [1]. Rising temperatures can favor the emergence of new diseases or parasites [2], [3], and can markedly modify host-parasite relationships [4]. Parasites are known to have detrimental effects on wildlife, either directly through blood loss or disturbance [5], or indirectly by enabling disease transmission [6]. Parasites can affect the health of their hosts at both the individual and population levels, triggering downstream effects on ecosystems and even human food security [7], [8]. Consequently, changes in parasite abundance and distribution have clear conservation and management implications. This appears especially true in high northern latitudes where climate warming is the most pronounced [9] and changes in host-parasite relationships have already been documented [4].

One parasite inhabiting polar environments that shows a response to climate warming is the seabird tick *Ixodes uriae* (Acari: Ixodidae, White 1852). This tick is a common ectoparasite which uses many colonial seabirds as host populations; it has a circumpolar distribution in both the Arctic and Antarctic [10]. The impact of ticks on seabirds can result in lower body condition, breeding success and even death [11], [12], [13], [14], [15], [16], [17]. The life cycle of the seabird tick is dependent on temperature and it cannot survive below −12°C, except during the egg stage [18]. Other closely related tick species (*I. ricinus and I. scapularis*) whose life cycles are also linked to temperature have already shown shifts in their distribution and abundance which have been linked to climate warming [19], [20], [21]. The distribution and abundance of *I. uriae* is thus likely to be affected by the ongoing warming of the Arctic, potentially generating detrimental effects on the host populations.

With the exception of one tick found in a kittiwake colony in 1999, *I. uriae* had not been observed on seabirds breeding in Spitsbergen until 2007 [22] despite intense fieldwork taking place on several seabird colonies in diverse locations within the region. Since 2007, ticks have been regularly observed in Spitsbergen but only on Brünnich’s guillemot (*Uria lomvia*) breeding at the Ossian Sarfjellet colony site, Kongsfjorden. Tick prevalence in that colony has shown strong inter-annual variations from near 0% to >35% prevalence. Here, I tested the prediction that tick prevalence at this Brünnich’s guillemot colony was positively linked to the previous winter’s temperature since ticks were first found in 2007, and tested for a negative effect of tick infestation on bird body condition and breeding success.

## Materials and Methods

### Study Species and Area

Brünnich’s guillemots are cliff-nesting seabirds that breed in dense colonies ranging from a few hundred to >100,000 individuals. Outside the breeding season, they spend most of their time at sea. They start egg laying at the end of May/early June, incubate a single egg for about four weeks and then feed their chick for approximately three weeks until the chicks disperse by jumping into the sea. Adult(s) stay with their chick at sea for an additional four to eight weeks, until chick independence [23].

This study took place at the Ossian Sarsfjellet colony, Kongsfjorden, Svalbard (78°55′ N, 12°26′ E) which comprises around 1100 breeding pairs. Capture/recaptures of breeding guillemots started in 2005 with 10 to 100 individuals being caught annually at several sites within the colony. Birds were captured using a noose pole, handled for approximately 10 minutes and then released. The first tick observation occurred in 2007 [22]. Ticks in this guillemot colony have been identified as *Ixodes uriae* [22], generally known as the seabird tick. *I. uriae* has three developmental stages (larvae, nymph, and adult) and the complete life cycle takes 3 to 4 years to complete. Larvae and nymphs from both sexes attach to the bird for single blood meal that lasts a few days and enables them to survive until the next life stage. As adults, only female ticks feed on the host before laying a single clutch and dying [24], [25].

**Figure 1:**
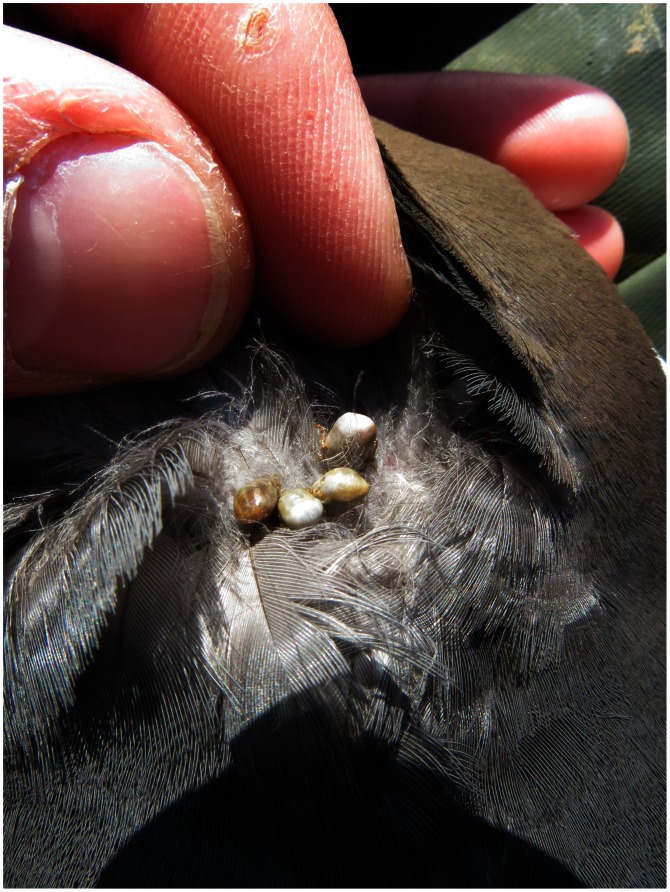
Engorged ticks *Ixodes uriae* on the neck of a Brünnich’s guillemot, Ossian Sarsfjellet colony, Svalbard (picture by D. Ruché).

Since 2007, all captured birds have been checked systematically for tick presence (visual inspection of the feet, cloacae, eyes and external plumage, with a manual check of the head and neck feathers; Fig. 1). This enables the detection of adults and nymphs as larvae are too small to be sampled in this way [26]. For most captured birds tick presence/absence (i.e., prevalence) was recorded but not the number of ticks (i.e., abundance); no distinction was made between adults and nymphs.

This study has been approved by the Governor of Svalbard (program number 361). Capturing and ringing Brünnich’s guillemots was not considered as an “animal experiment” and did not require any permit from the Norwegian Animal Care Authority (Forsøksdyrutvalget).

### Study Design and Analyses

To assess the relationship between winter temperature and tick prevalence, I used temperature data from the Ny Ålesund airport weather station (mean monthly temperature between January and March, available at http://www.met.no). This station is located in the same fjord as the study colony, 12 km east (78°55′28″N 11°55′42″E). The effect of winter temperature on the following summer’s tick prevalence was assessed by linear regression (glm with a normal error distribution, data not transformed; Shapiro-Wilk normality test on the residuals: p = 0.60).

To assess the effects of tick presence on bird body condition, I used bird body masses (adjusted for body size, capture date and year) collected from 2008 to 2012 (n = 225 birds). Data from 2007 were not included because the identities of the birds found with ticks were, unfortunately, not recorded. I used a multiple linear regression (glm with a normal error distribution; Shapiro-Wilk normality test on the residuals: p = 0.99). Brünnich’s guillemots show a sharp decrease in body mass after hatching [27]; breeding status of birds (on egg or on chick) could thus potentially affect the apparent relationship between tick prevalence and bird body condition if tick prevalence also varies with bird breeding status. Indeed, an apparent decrease in bird body mass could be wrongly interpreted as a detrimental effect of tick presence, where it is simply due to a decrease in mass following hatching that parallels an increase in tick prevalence as the season progresses. However, tick prevalence showed no relationship with capture date, at least in 2012 when sufficient data were available for such a test (capture date effect on tick presence: p = 0.16). Furthermore, the same effect of tick presence on bird body mass was obtained when bird breeding status was included as a covariate in the model (n = 102 birds with known breeding status at capture).

Finally, I assessed the effect of tick presence on bird breeding success by using data collected in 2012 (n = 41 birds from 41 different nests with known breeding success). The monitoring of individual breeding success started in 2011 but in that year, only 1 of 80 birds captured had ticks. Breeding success was estimated by chick survival until 15 days of age. Chicks usually leave the colony around 21 days of age, but due to field constraints I was only able to assess chick survival until 15 days after hatching. The effect of tick presence on bird breeding success in 2012 was assessed by logistic regression (glm with a binomial distribution; no overdispersion,  = 1.05). All analyses were performed with *R* software [28].

## Results

**Figure 2:**
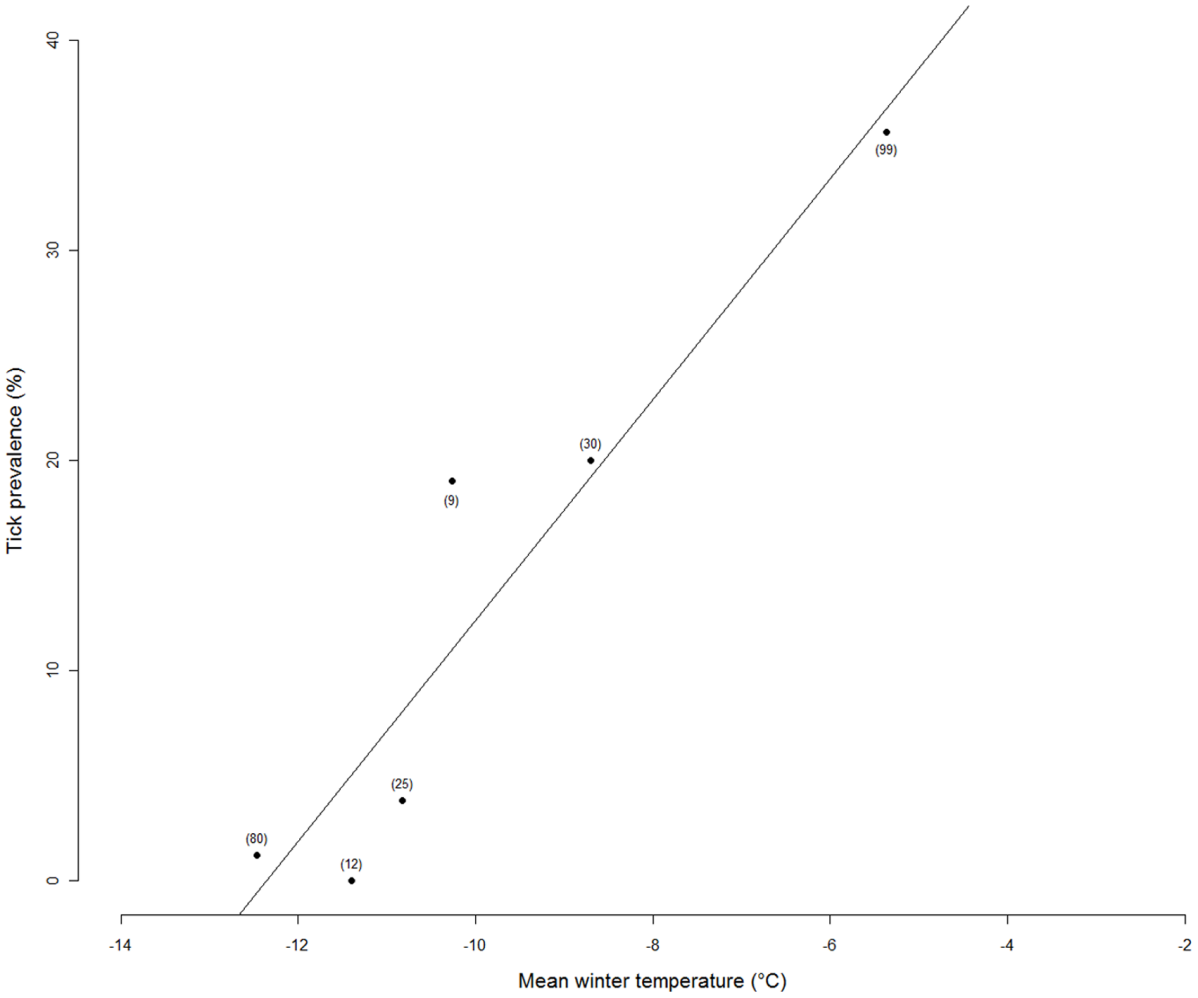
Winter temperature and tick prevalence. Relationship between mean winter temperature in Kongsfjorden (average monthly temperature between January and March) and the prevalence of tick infestation on Brünnich’s guillemot (*Uria lomvia*) at the Ossian Sarsfjellet colony, Svalbard archipelago (R^2^ = 0.89, p = 0.005). The total number of guillemots caught each season is noted in brackets near each circle. The relationship remains marginally significant when the warmest year was excluded (R^2^ = 0.73, p = 0.067).

Mean winter temperature had a significant effect (p = 0.005) on tick prevalence at the Ossian Sarsfjellet colony (Fig. 2). The slope of this effect was 5.2, resulting in an average 1°C increase in winter temperature that was associated with a 5% increase in the prevalence of ticks the following summer. Winter temperature explained 89% of the variance in tick prevalence from 2007–2012. The 2012 winter was exceptionally warm (−5.4°C; i.e., >5°C above the average for the previous decade) and tick prevalence in the following summer was very high as well (>35%). However, the relationship between winter temperature and tick prevalence was not only driven by this single year because the relation remained nearly significant after excluding the year 2012 (R^2^ = 0.73 and p = 0.067). Moreover, this relationship was not driven by a common trend in both data series (i.e., neither winter temperature nor tick prevalence showed a significant trend over the study period).

I did not find any effect of the presence of ticks on guillemot body condition. The effect of tick presence (binary variable: presence/absence) on body mass was not significant (*t = *−*0.62, p = 0.53*) after taking into account body size (i.e., head length: *β = 5.31±1.32, t = 4.03, p<0.001*), date of capture (*β = *−*1.88±0.52, t = *−*3.61, p<0.001*) and year (*p = 0.33*). The average mass (±SE) was 1000±13 g and 984±5 g for birds with and without ticks, respectively. Similarly, I did not find any effect of tick presence on breeding success in 2012 (effect of tick presence: z = 0.52, p = 0.61). The average breeding success (±SE) was 0.83±0.11 (n = 12) and 0.76±0.08 (n = 29) for birds with and without ticks, respectively.

## Discussion

The seabird tick is a major parasite of seabirds breeding at high latitudes [29]. Tick survival and activity are dependent on ambient temperature [18], [30] so its abundance, distribution and impact on wildlife are likely to increase due to ongoing climate warming. This parasite has only recently been observed in Spitsbergen [22]; it may have newly arrived in the archipelago, or its abundance may have increased [22]. As discussed in [22], climate warming is a plausible explanation for changes in tick abundance or distribution. My results support this climate warming scenario. Within the last 6 years, the prevalence of tick infestation within the Ossians Sarsfjellet guillemot colony was strongly related to the previous winter’s temperature. This single environmental variable explains almost 90% of the variation in the average tick prevalence. Moreover, the effect of winter temperature was strong, with a 1°C increase in January to March average temperature associated with a 5% increase in tick prevalence. Warming in the Arctic is projected to range from 2°C to 9°C by the year 2100, depending on the model and forcing scenario [9], and this warming is expected to be highest in winter. Based on those climatic predictions, the average tick prevalence in the Ossian Sarsfjellet guillemot colony could increase by 10 to 45% by the year 2100.

The direct impact of ticks at individual and population levels depends mostly on tick abundance. It was not systematically recorded throughout this study colony, but fewer than five ticks were generally found on individual birds. However, in 2007 one adult Brünnich’s guillemot was found dead in the fjord with more than 200 ticks on it (G.W. Gabrielsen, pers. comm.). It is therefore not surprising that there was no detectable effect of tick presence on guillemot body condition and breeding success given that such a low number of ticks per bird is unlikely have direct effects such as anemia [31]. Considering that tick meal size is estimated to be around 130 mg for an adult and 10 mg for a lymph [32], five ticks per bird, either adults or nymphs, cannot cause anemia in a 1 kg bird such as the Brünnich’s guillemot. Moreover, as breeding success is influenced by both parents (both incubate and feed the chick), data on tick prevalence in both parents would be needed to adequately test for a tick effect on guillemot breeding success.

However, although there were no detectable detrimental direct effects of ticks on breeding adults, they may still have the potential to affect Brünnich’s guillemots through disease transmission [10] or through effects on guillemot chicks. Indeed, chicks may be more sensitive to ectoparasites than adults [33], with potential long-term population dynamic consequences.

Brünnich’s guillemots are declining within the archipelago (Descamps et al., unpublished results) and an increase in ectoparasite load may represent an additional stressor on birds already challenged by changes in their marine environment. The presence and abundance of ticks in Svalbard seabird colonies thus deserves detailed monitoring if we are to follow changes in the tick distribution, abundance and impact on bird populations in relation to the warming of the Arctic.
